# Occlusion facial expression recognition based on feature fusion residual attention network

**DOI:** 10.3389/fnbot.2023.1250706

**Published:** 2023-08-17

**Authors:** Yuekun Chen, Shuaishi Liu, Dongxu Zhao, Wenkai Ji

**Affiliations:** School of Electrical and Electronic Engineering, Changchun University of Technology, Changchun, China

**Keywords:** occluded facial expression recognition, feature fusion network, multi-scale module, local attention module, attention mechanism

## Abstract

Recognizing occluded facial expressions in the wild poses a significant challenge. However, most previous approaches rely solely on either global or local feature-based methods, leading to the loss of relevant expression features. To address these issues, a feature fusion residual attention network (FFRA-Net) is proposed. FFRA-Net consists of a multi-scale module, a local attention module, and a feature fusion module. The multi-scale module divides the intermediate feature map into several sub-feature maps in an equal manner along the channel dimension. Then, a convolution operation is applied to each of these feature maps to obtain diverse global features. The local attention module divides the intermediate feature map into several sub-feature maps along the spatial dimension. Subsequently, a convolution operation is applied to each of these feature maps, resulting in the extraction of local key features through the attention mechanism. The feature fusion module plays a crucial role in integrating global and local expression features while also establishing residual links between inputs and outputs to compensate for the loss of fine-grained features. Last, two occlusion expression datasets (FM_RAF-DB and SG_RAF-DB) were constructed based on the RAF-DB dataset. Extensive experiments demonstrate that the proposed FFRA-Net achieves excellent results on four datasets: FM_RAF-DB, SG_RAF-DB, RAF-DB, and FERPLUS, with accuracies of 77.87%, 79.50%, 88.66%, and 88.97%, respectively. Thus, the approach presented in this paper demonstrates strong applicability in the context of occluded facial expression recognition (FER).

## 1. Introduction

Facial expression recognition (FER) has emerged as a critical research direction in the field of artificial intelligence due to the significant role facial expressions play in daily interpersonal communication. FER holds potential applications across diverse fields, including intelligent tutoring systems, service robots, and driver fatigue detection (Poulose et al., 2021a,b). As a result, it has garnered increasing attention in the field of computer vision in recent years.

FER methods can be categorized into two types depending on the scenario: studies conducted in a controlled laboratory environment and studies conducted outside the laboratory in an uncontrolled environment. In controlled environments, the small sample size of the collected data affects the model's feature learning. To overcome this, some researchers propose a new encoder-decoder structure that generates various facial expression images, effectively expanding the sample size (Zhang et al., 2018). Furthermore, Xue et al. (2021) proposed the TransFER model, investigating the relationship between global Transformer-extracted features and local CNN-extracted features. This enhances feature learning and improves model performance. However, these approaches primarily rely on studies conducted on laboratory datasets, such as CK+ (Lucey et al., 2010), MMI (Valstar and Pantic, 2010), and OULU-CASIA (Zhao et al., 2011). Despite achieving high accuracy on these datasets, FER methods exhibit poor performance in uncontrolled environments. To address this, some researchers have tackled class imbalance and label noise issues in datasets by utilizing techniques like data augmentation and auxiliary datasets (Wang et al., 2018). Network interpretability studies demonstrate that models can prioritize relevant facial expression features, resulting in more accurate emotion detection (Kim et al., 2021). Additionally, the noisy labeling problem in real-world datasets can be mitigated by introducing a probabilistic transformation layer (Zeng et al., 2018). The above methods are investigated on expression datasets in uncontrolled environments. However, FER still faces challenges when the face is partially occluded by objects like sunglasses, scarves, masks, or other random items that frequently occur in real images or videos.

**Figure 1:**
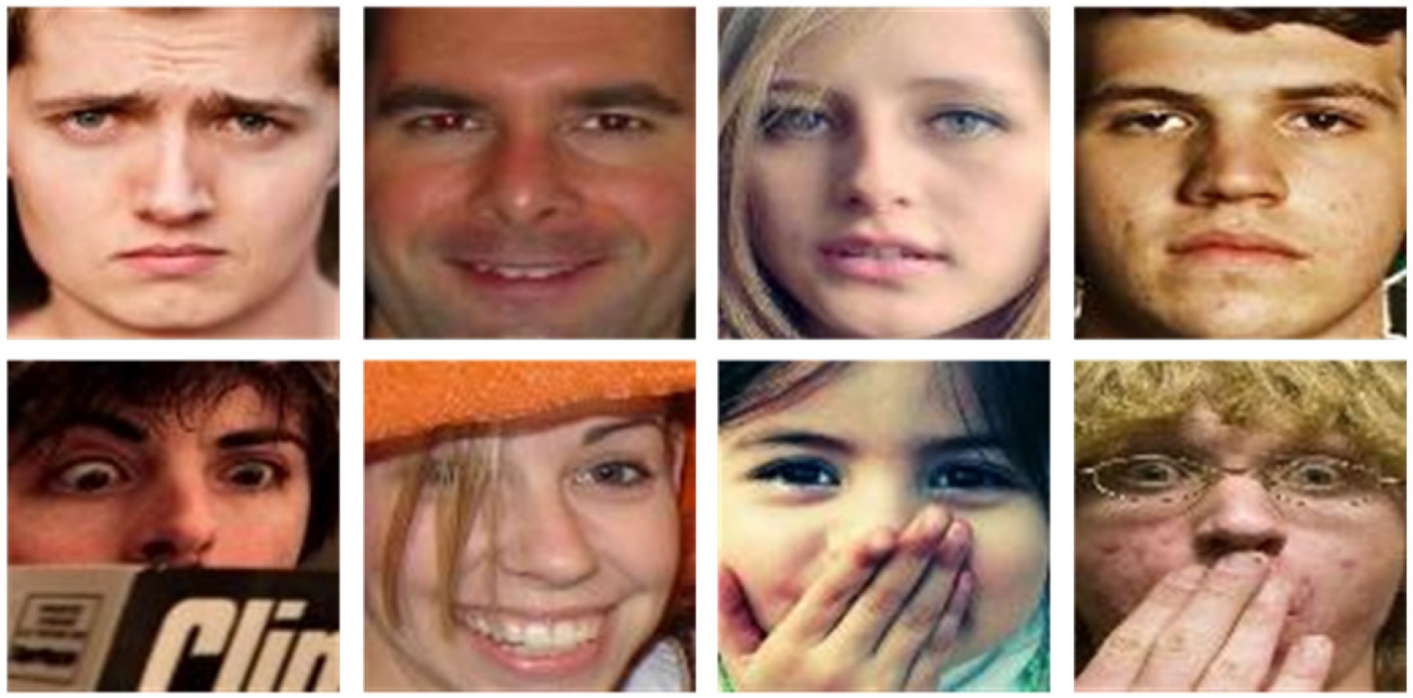
Some examples of images from the RAF-DB dataset, where the first row comprises non-occluded expression images and the second row comprises occluded expression images.

Addressing the facial occlusion problem is crucial for improving the performance of FER models in real-world environments. As shown in Figure 1, the occlusion problem leads to a large spatial change in the appearance of the face. To tackle this issue, certain researchers have suggested utilizing deep CNN networks for solving the occlusion problem. Specifically, two CNN networks are trained from a global perspective using occluded and non-occluded face images. The non-occluded face images are utilized as privileged information for fine-tuning the occluded expression recognition network. This approach (Pan et al., 2019) significantly reduces occlusion interference and enhances network performance. However, the drawback of this FER algorithm is its focus solely on global features, neglecting the crucial local detail features that play a vital role in expression discrimination. Therefore, regarding the occlusion FER problem, certain researchers suggested a method based on local keypoint localization (Wang K. et al., 2019), effectively capturing crucial local facial features. However, choosing the appropriate local regions remains a key issue. To address this, researchers employed three local region generation schemes: fixed position selection, random selection, and labeled keypoint selection. This approach significantly enhances the performance of the occlusion FER model. An alternative method for keypoint selection involves choosing 24 facial keypoints to define 24 key local regions. Subsequently, an attention network is employed to extract features from each region, allowing better focus on important local features. This approach (Li et al., 2018) offers a viable solution to the occlusion FER problem. Nonetheless, the localization-based approach has a drawback of neglecting global information, which limits its overall ability in expression discrimination. Consequently, the effective combination of global and local features is paramount in addressing the occlusion FER problem.

To solve the above issues, a feature fusion residual attention network aiming to enhance feature robustness is proposed. In convolutional neural networks (CNNs), deep convolutions exhibit a broader receptive domain and encompass richer semantic features, whereas shallow convolutions have a narrower receptive domain and capture rich profile features. However, deep convolutions are susceptible to occlusion (Proverbio and Cerri, 2022). To address this, this paper employ multi-scale modules to extract features from diverse receptive domains, thereby enhancing the diversity and robustness of global features. Additionally, this paper design local attention modules to extract local features, mitigating occlusion interference. To learn both global multi-scale and local features, this paper employed a two-branch network. The first branch utilized the multi-scale module, while the second branch divided the extracted feature maps into multiple non-overlapping local feature maps, which were then processed using the attention mechanism. Finally, the processed features were fused. The main contributions of this paper can be summarized as follows:

1. Feature fusion residual attention network (FFRA-Net), a simple and effective FER network, is proposed to address the challenge of facial occlusion by enhancing the diversity of expression features through feature fusion.
2. The multi-scale module extracts features at different scales from the feature map, thereby reducing the sensitivity of deep convolutions to occlusion. Additionally, the local attention module focuses on local salient features and mitigates occlusion interference.

The remainder of this paper is structured as follows. Section 2 provides a review of relevant literature. Subsequently, the proposed approach is presented in Section 3. Section 4 presents the experimental results for both obscured and non-obscured expression datasets. Additionally, visualizations are provided to further validate the proposed method. Section 5 summarizes the findings.

## 2. Related work

### 2.1. Deep convolutional FER

In recent years, researchers have made significant progress in FER by proposing numerous methods based on deep CNNs. However, deep learning-based FER often disregards domain-specific knowledge related to facial expressions. To tackle this issue, Chen et al. (2019) introduced a framework for FER that leverages prior knowledge by utilizing the distinctions between neutral expressions and other expressions to train the network. Moreover, head pose variation poses a common challenge in expression recognition. To tackle this issue, Marrero-Fernández et al. (2019) propose an end-to-end architecture with an attention mechanism that rectifies facial images to improve expression classification. Due to the subtle variations in expressions, the issue of inter-class similarity in expression datasets becomes crucial. To address this, Wen et al. (2021) proposed attention distraction networks. The aforementioned methods primarily concentrate on datasets obtained in controlled environments, where facial images are predominantly frontal. Consequently, the model's performance suffers when it comes to recognizing facial expressions in uncontrolled environments.

To differentiate between uncertain and blurred expression images in uncontrolled environments, Pu et al. (2020) proposed an expression recognition framework based on facial action units. The framework incorporates an attention mechanism that dynamically focuses on significant facial actions. To quantify these uncertainties, Zhang et al. (2021) proposed a relative uncertainty method that assigns weights based on uncertainties, fuses facial features, and introduces a new uncertainty loss. She et al. (2021) introduced a multi-branch learning network to address the label ambiguity problem in FER. The method enhances the ability to explore and capture the underlying distribution in the label space. Furthermore, the expression dataset faces challenges posed by pose variation and identity bias. To tackle these challenges, Wang C. et al. (2019) proposed an adversarial feature learning method. The gesture discriminator and identity discriminator classify gestures and identities based on the extracted feature representations, respectively. Similarly, Chen and Joo (2021) presented a FER framework based on facial action units. The framework integrates a triple loss into the objective function, leading to improved expression classification accuracy. Despite the impressive performance of the aforementioned methods on uncontrolled environment data, the task of masking FER remains challenging.

### 2.2. Occluded FER

Considering the limited availability of large-scale occluded expression datasets, Xia and Wang (2020) proposed a stepwise learning strategy for occluded FER models. The distribution density in the feature space is first used to measure the complexity of the non-occluded data, thus guiding the distribution of the occluded expression features to converge to the distribution of the non-occluded expression features. In a similar vein, Pan et al. (2019) presented a novel method for occluded FER that leverages non-obscured face image information. This approach aims to align the distribution of learned occluded face image features with the distribution of non-occluded face image features. Nonetheless, the aforementioned methods rely on global features. In occlusion expression recognition, global features are susceptible to the influence of occlusion, leading to reduced accuracy in expression recognition. To overcome this challenge, Wang K. et al. (2019) introduced a network based on local region attention. Additionally, they proposed a region bias loss to assign weights to local region attention. Xue et al. (2022) proposed a dedicated attention mechanism for FER networks. The proposed model selectively focuses on the most relevant expression features while disregarding irrelevant features, thereby avoiding undue emphasis on occlusion or other noisy regions. The aforementioned approach based on local features effectively addresses the occlusion problem. However, it overlooks global information and possesses limited discriminative ability for expression as a whole.

Hence, it is crucial to consider both global and local features for effective occluded expression recognition. Ding et al. (2020) introduced an adaptive depth network for recognizing occluded facial expressions. Initially, global features are extracted using the ResNet-50 backbone network. Subsequently, the network is partitioned into two branches. Each branch is further divided into multiple sub-regions, with each sub-region independently predicting expressions. Finally, strategy fusion is conducted to obtain the final classification results. Zhao et al. (2021) presented an expression recognition network capable of learning global and local features. This network effectively mitigates the deep network's sensitivity to occlusion and autonomously attends to local key information. Finally, the same policy fusion is employed to derive the results. Nevertheless, the policy fusion approach is prone to overfitting as the network deepens and shows poor performance when trained on certain realistic occlusion data.

## 3. Proposed method

**Figure 2:**
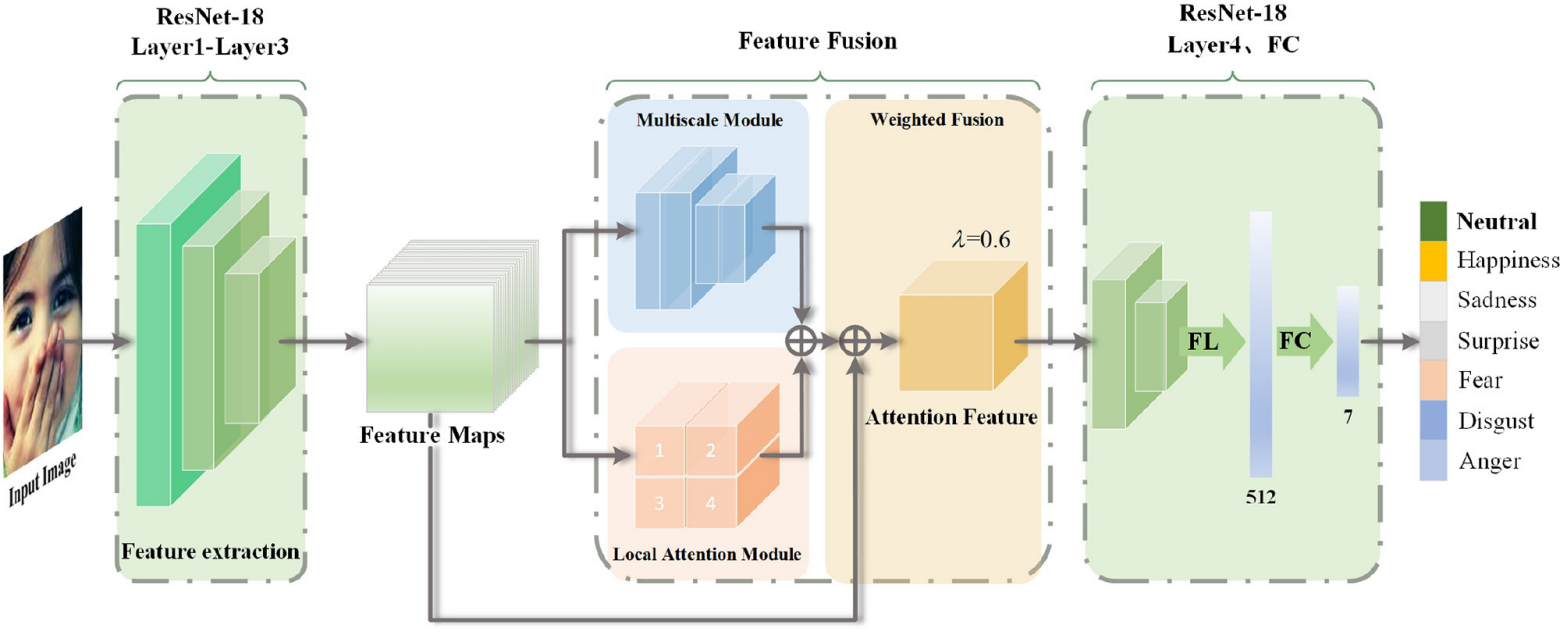
The model structure employed in this paper. The method includes the backbone network ResNet-18, along with a multi-scale module, a local attention module, and a weighted fusion module. Here, Input image from the RAF-DB dataset, λ = 0.6 represents the weight assigned to local features, FL indicates the tiling operation, and FC refers to the fully connected network.

FFRA-Net is a feature fusion network designed to address the recognition of obscured facial expressions. The method comprises a multi-scale module, a local attention module, a feature fusion module, and a residual link. The backbone network chosen for this purpose is ResNet-18 (He et al., 2015). Figure 2 illustrates the structure of FFRA-Net. Initially, the feature preextractor captures the intermediate facial expression features, which are obtained from the first three convolutional stages of ResNet-18. Then, a two-branch network is used to process the acquired intermediate feature maps into the multiscale module and the local attention module, respectively, allowing the model to obtain both global and local expression features. Subsequently, the model enters the feature fusion phase, where a weighted fusion approach is applied to assign specific weights to the feature mappings from the two branches. These weighted features are then directly summed. Meanwhile, it is then added with the original intermediate feature map to form a residual connection, and finally a global and local attention feature map is obtained. Finally, this feature map proceeds to the last convolutional stage of ResNet-18, followed by fully connected layers for deriving the classification results.

### 3.1. Multi-scale module

**Figure 3:**
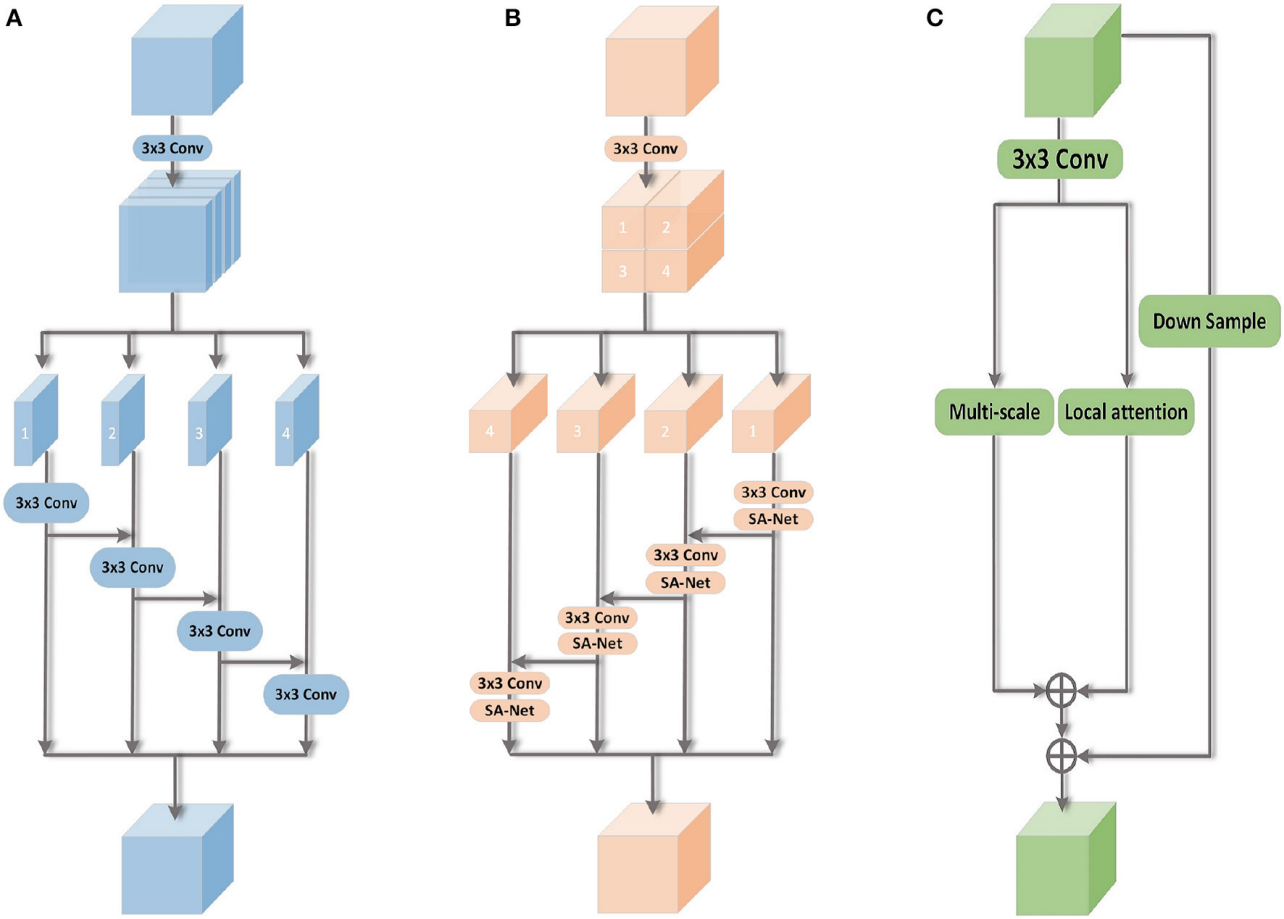
FFRA-Net uses three types of modules. Multi-scale module, local attention module, and feature fusion module. **(A)** Multi-scale. **(B)** Local attention. **(C)** Feature fusion.

Multi-scale modules are widely used in computer vision for processing visual information across different scales (Gao et al., 2019; Ma and Zhang, 2023). It is widely used in many tasks, including target detection and image segmentation. Typically, the multi-scale module divides the feature map into multiple subregions of different scales in the spatial dimension, processing each subregion individually. However, this approach is primarily applicable to visual tasks like target detection and image segmentation. Occluded expression recognition is influenced by occlusions, leading to the absence of certain semantic information. To compensate for this deficiency, there is a need for more comprehensive and diverse global features. To tackle this issue, a novel multi-scale image classification module is proposed (Figure 3A). The feature map is divided into multiple sub-feature maps along the channel dimension, enabling the extraction of a broader range of global expression information.

The objective of this method is to learn multi-scale features within the feature map while ensuring that the feature subsets encompass a wider range of scale information. Specifically, the feature mapping *X* is obtained through feature pre-extraction. Next, the module partition *X* into *n* feature map subsets along the channel axis, denoted as *X*_*i*_, with *i*∈{1, 2, …, *n*} representing the index. Each feature subset *X*_*i*_ has the same spatial size as the feature map *X* but contains only 1/*n* channels. Subsequently, a 3 × 3 convolution is applied to each *X*_*i*_, yielding the output denoted as $P_{i}^{ms}$, while $Y_{i}^{ms}$ represents the output after fusion of each sub-feature. Therefore, the expression for each output $Y_{i}^{ms}$ can be defined as follows:


(1) $$Y_{i}^{ms}=\{\begin{array}{ll} P_{i}^{ms}\left(X_{i}\right) & i=1\\ P_{i}^{ms}\left(X_{i}+Y_{i-1}^{ms}\right) & 1<i⩽n \end{array}$$

Equation 1 demonstrates that each output $Y_{i}^{ms}$ encompasses a distinct number and scale of subset features. In order to obtain a more diverse collection of global features, the module concatenate all the $Y_{i}^{ms}$ outputs along the channel dimension. However, increasing the value of *n* results in features containing more scale information, which in turn increases model complexity and computational overhead. Taking these factors into consideration, *n* is set to 4 in this module to optimize the performance of the model.

The multi-scale convolution captures comprehensive and detailed global information in the feature map, thereby reducing the sensitivity of deep convolution to occlusion. Compared to the traditional ResNet-18 network, this network selectively attends to the facial regions related to expression while disregarding occluded regions, thus effectively addressing the issue of facial occlusion.

### 3.2. Local attention module

The local attention module, commonly used in computer vision, utilizes the attention mechanism to capture essential information from images. The attention mechanism, similar to human vision, assigns weights to channels or spatial domains through automatic learning. This enables the neural network to focus on important regions and disregard others. In occlusion-based FER, a portion of the facial image is obscured by an occluder, leading to a loss of discriminative ability in the occluded region's features. Based on this feature, a novel local attention module (Figure 3B) is proposed. This module significantly enhances the model's perceptual capability.

Local features play a crucial role in occlusion FER. However, previous methods often employ face tagging or random cropping to divide faces into multiple local regions in order to extract effective local features. but these methods may result in redundancy of features and increase in computational overhead. To solve this issue, the intermediate feature maps are divided into non-overlapping local feature maps, aiming to enable each local feature map to autonomously focus on local key features using attention mechanism. Therefore, after 3 × 3 convolution of the feature maps obtained by feature pre-extraction, the module divide the extracted feature map *S* into several local feature maps *S*_*i*_ along the spatial axis, where *i*∈{1, 2, …, *m*}. Each *S*_*i*_ undergoes a 3 × 3 convolution, resulting in a feature map denoted as *F*∈ℝ^*H*×*W*×*C*^. Shuffle Attention (SA) mechanism was subsequently used as the attention network (Zhang and Yang, 2021). The SA module divides the input feature map into *G* sub-feature maps evenly across the channel dimension, where *G* is set to 8. Subsequently, each sub-feature map is evenly divided into two feature maps along the channel dimension. Then, the SA module calculates the channel and spatial attention weights for each of the two feature maps successively, focusing on the channel and spatial dimensions, respectively. Subsequently, the attention weights are multiplied with the original feature maps to generate attention maps in both dimensions. As shown in Equations 2 and 3, these two attention maps are then combined, and the same process is repeated for the remaining sub-feature maps. The interaction between each sub-feature graph is achieved through the channel shuffle operation. Channel shuffle involves randomly rearranging the original channel order of the feature map before their combination. Finally, an attention graph with the same shape as the input feature graph is generated. In our network, each $F_{i}\in {{\mathbb{R}}^{H\times W\times C}}^{/G}$ (where *i*∈{1, 2, …, *G*}) is further divided into ${F_{i}}_{j}\in {{\mathbb{R}}^{H\times W\times C}}^{/2G}$ (where *j*∈{1, 2}), and the attention network takes _*F*_*i*_*j*_ as input. It calculates a one-dimensional channel attention weight map $M_{c}\in {\mathbb{R}}^{1\times 1\times C}$ and a two-dimensional spatial attention weight map $M_{s}\in {\mathbb{R}}^{H\times W\times 1}$ for element-level multiplication denoted by ⊗, and outputs the result as *F*_*r*_ after stitching the sub-attention maps (*F*_*r*_). Therefore, the attention network can be expressed as follows:


(2) $$\begin{array}{l} \begin{array}{c} F_{ri}=\left[\left(M_{s}\left(F_{ij}\right)⊗F_{ij}\right),\left(M_{c}\left(F_{ij}\right)⊗F_{ij}\right)\right] \end{array} \end{array}$$

(3) $$\begin{array}{l} \begin{array}{c} F_{r}=\left[{\text{F}}_{\text{r}1},\cdots \;,{\text{F}}_{\text{rG}}\right] \end{array} \end{array}$$

Let the output of the 3 × 3 convolutional and attentional network be denoted as $P_{i}^{la}$, and the output after feature fusion as $Y_{i}^{la}$. Thus, each output can be expressed as follows:


(4) $$Y_{i}^{la}=\{\begin{array}{ll} P_{i}^{la}\left(S_{i}\right) & i=1\\ P_{i}^{la}\left(S_{i}+Y_{i-1}^{la}\right) & 1<i⩽n \end{array}$$

Based on Equation 4, each output comprises varying numbers and sizes of local features. To obtain a wider range of diverse local features, the module concatenate all the outputs along the spatial dimensions. In this study, *m* is set to 4, which aligns better with the characteristics of masked expression images and guarantees improved model performance.

### 3.3. Feature fusion module

In computer vision, a feature fusion module is employed to integrate information from diverse feature types, enhancing the performance of vision tasks. To maintain a balance between the significance of multi-scale and local attention features, weights are incorporated into the feature fusion module. Figure 3C illustrates the integration of global and local information within this module, resulting in improved model performance. Furthermore, to enhance the network's expressive capacity, the module establish residual connections between the input and output features. This enables the network to more effectively capture image details and contextual information. Here, the original input features are denoted as *X*, the outputs of the multi-scale and local attention modules as $Y_{i}^{ms}$ and $Y_{i}^{la}$, respectively, and the output of the final feature fusion module as *X*. Therefore, it can be expressed as:


(5) $$\begin{array}{l} \begin{array}{c} Y=\lambda Y_{i}^{\text{la}}+\left(1-\lambda \right)Y_{i}^{\text{ms}}+X \end{array} \end{array}$$

In Equation 5, λ represents a hyperparameter that controls the relative significance of the multi-scale and local attention modules. It is demonstrating experimentally that the model achieves the best performance when λ is set to 0.6.

## 4. Experiment

This section describes the data set used and the data processing procedures. And the details of the experimental setup are presented. Then, the experimental results are presented, including the results of the ablation experiments, the determination of the feature fusion weights, the visualization of the CAM, and the results of the partial confusion matrix. Last, the method of this paper is compared with other methods, and the experimental results are comprehensively analyzed.

### 4.1. Datasets

RAF-DB (Li and Deng, 2019): RAF-DB, a real-world expression dataset, comprises 29,672 facial expression images. These images were independently annotated by approximately 40 annotators. The experiments in this paper utilized a single tag provided by RAF-DB. The dataset consists of 15,339 expression images, encompassing six basic expressions (happy, surprised, sad, angry, disgusted, and fearful), as well as neutral expressions. Out of these, 12,271 images were allocated for training, while 3,068 were allocated for testing.

FERPLUS (Barsoum et al., 2016): FERPLUS is an extension of FER2013, a large-scale dataset collected using the Google Image Search API. The dataset comprises 28,709 training images, 3,589 validation images, and 3,589 test images. It was re-labeled by 10 annotation workers to include six basic expressions (happy, surprised, sad, angry, disgusted, and fearful), as well as neutral and contemptuous expressions.

**Figure 4:**
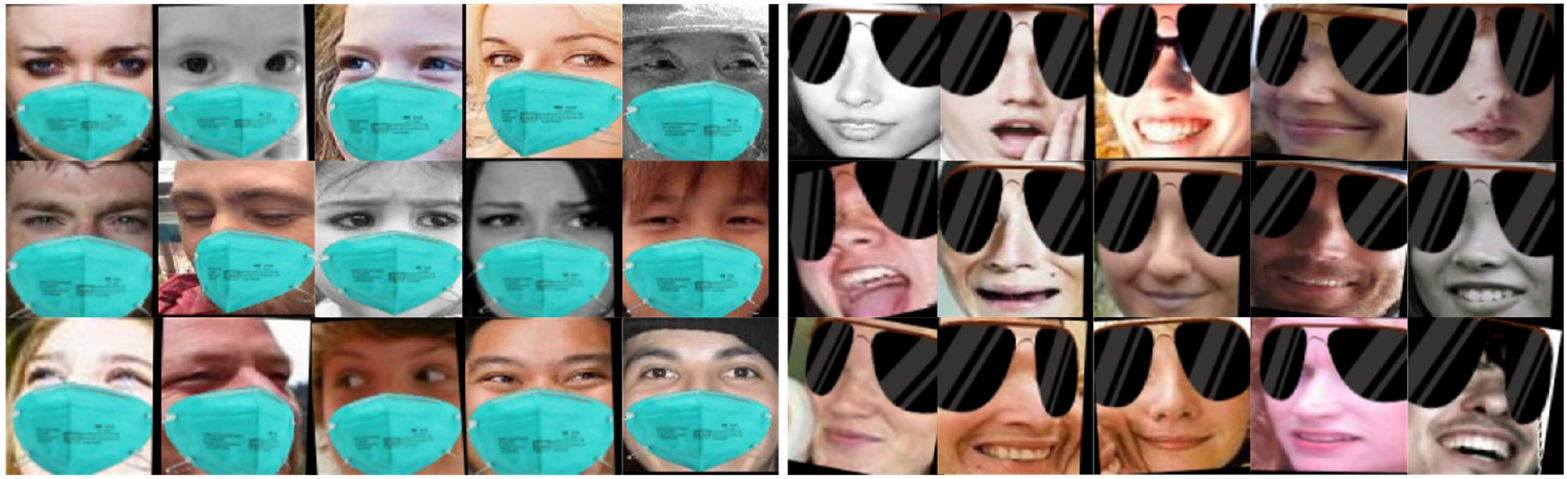
Some image examples of FM_RAF-DB and SG_RAF-DB dataset.

FM_RAF-DB and SG_RAF-DB: To evaluate the performance of our proposed FER model under realistic occlusion conditions, two occlusion representation datasets were created based on RAF-DB: FM_RAF-DB and SG_RAF-DB. Using face detection (Deng et al., 2020), these datasets simulate both cases of faces wearing masks and sunglasses. The masked face method used, specifically, marks the key points of the face and selects the key points around the eyes and mouth. The method then uses a bionic matrix and a bionic transformation calculation to place the mask image and the sunglasses image in their respective positions (refer to Figure 4). These two datasets better simulate the facial occlusion in real scenes, allowing a more accurate evaluation of the performance of our proposed FER model.

### 4.2. Implementation details

For all datasets, official face-aligned samples are used. The input images of RAF-DB and FERPLUS datasets were cropped to a size of pixels, respectively. In this study, the ResNset-18 network was chosen as the backbone network and the experimental code was implemented using the PyTorch framework. The training was conducted on an NVIDIA RTX-3090 GPU. In this study, a pre-trained ResNet-18 model obtained by training on the MS-Celeb-1M dataset was utilized. The optimizer used for training is the Adam optimizer with a batch size of 128 and an initial learning rate of 0.0001. To achieve the best results, the model in this paper was trained on all datasets for 200 epochs.

### 4.3. Ablation studies

In order to assess the effectiveness of FFRA-Net, this section performed ablation experiments on the FM_RAF-DB and SG_RAF-DB datasets. The experimental results encompass the selection of feature fusion strategy, the value of the weight hyperparameter, the impacts of the multi-scale module and local attention module on the model, as well as CAM visualization.

#### 4.3.1. Selection of the feature fusion strategy

In this subsection, different fusion strategies are experimented on the SG_RAF-DB dataset. Table 1 presents the comparison results of three feature fusion strategies: splicing fusion, summing fusion, and weighted fusion. Splicing fusion involves concatenating two feature maps along the channel dimensions and subsequently fusing the information from all channels through convolution. Additive fusion directly adds the feature maps obtained from two branches to create a combined feature map. Weighted fusion assigns specific weights to the feature maps of different branches based on additive fusion and then adds them together. In this study, the weight for the local attention module is empirically set to 0.6, as verified in subsequent subsections. The results demonstrate that weighted fusion is a more suitable fusion method.

**Table 1:** Evaluating various fusion strategies on the SG_RAF-DB dataset.

**Fusion strategies**	**Acc.(%)**
Concate feature fusion	76.69
Add feature fusion	77.87
Weighted feature fusion	79.50

#### 4.3.2. The value of the weight hyperparameter λ

**Figure 5:**
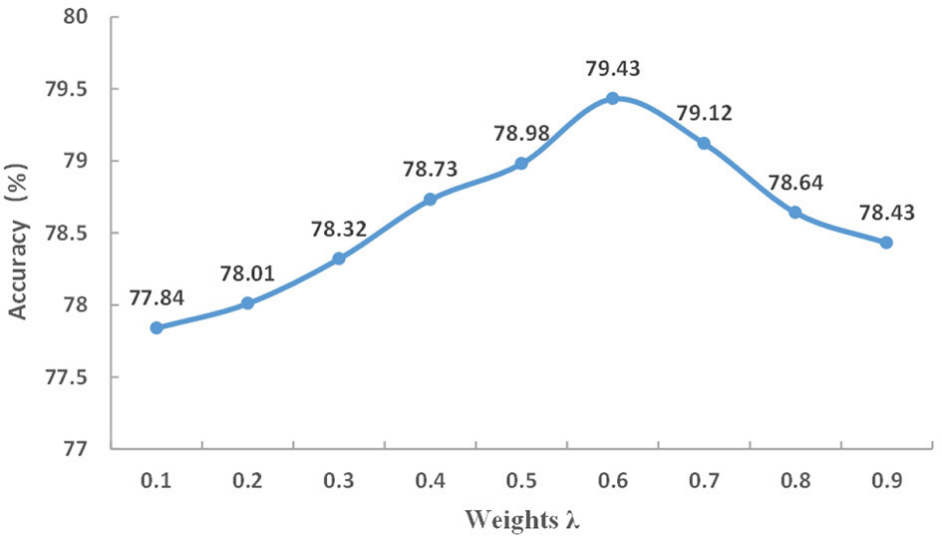
Evaluation of different λ values on the SG_RAF-DB dataset.

To balance the importance of multi-scale modules and local attention modules, λ is used as a hyperparameter. The local attention weight is set to λ, and the weight of the multiscale module is set to 1−λ. This experiment investigate different values of λ ranging from 0.1 to 0.9 to examine its effect on FFRA-Net, and the results are presented in Figure 5. When λ is set to 0.6, the weight of the local attention branch is slightly higher than that of the multi-scale branch, leading to the model achieving the best performance.

#### 4.3.3. Effects of multi-scale modules and local attention modules

An ablation analysis was conducted to verify the effectiveness of the multi-scale module and the local attention module in FFRA-Net. The results in Table 2 demonstrate that using either the multi-scale module or the local attention module alone yields higher accuracy compared to the baseline accuracy. Moreover, the local attention module exhibits greater usefulness than the multi-scale module. Ultimately, the model achieved the best performance by employing both modules and integrating their features.

**Table 2:** Evaluation of multi-scale and local attention modules in networks on the FM_RAF-DB and SG_RAF-DB datasets.

**Multi-scale**	**Local attention**	**FM_RAF-DB**	**SG_RAF-DB**
-	-	75.98%	77.44%
✓	-	76.86%	78.62%
-	✓	77.74%	79.37%
✓	✓	77.87%	79.50%

**Figure 6:**
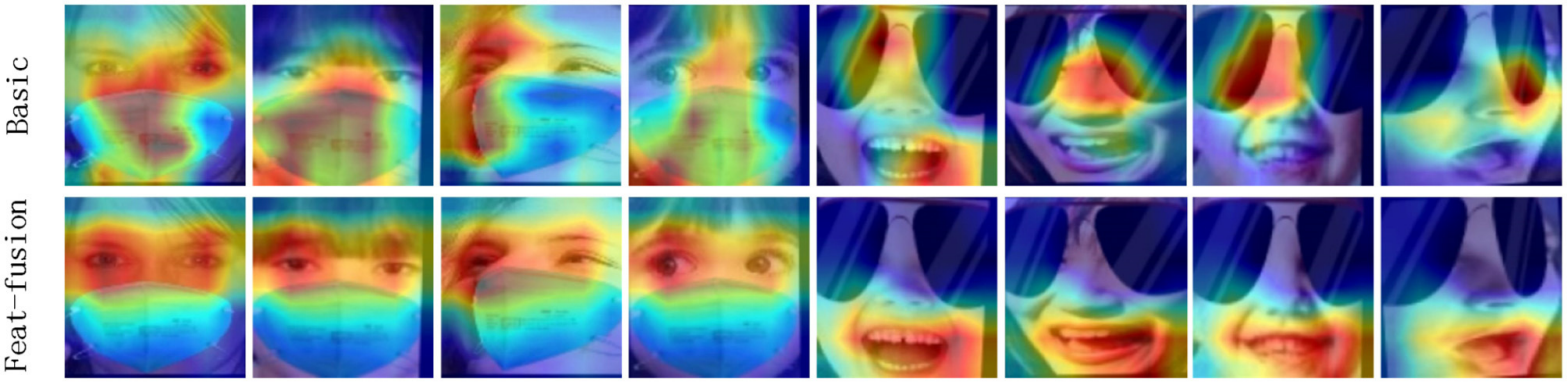
Feature fusion and CAM visualization of ResNet-18. Images are from the test set of FM_RAF-DB and SG_RAF-DB datasets.

To provide a clearer understanding of the effect of the feature fusion module, the study conducted CAM visualization (Zhou et al., 2015) to validate its performance. Figure 6 displays the visualization results of the baseline and feature fusion modules in the first and second rows, respectively. In comparison to the traditional ResNet-18, the CAM results obtained with feature fusion direct the network's attention toward locally significant regions. For the first four images where faces are covered by masks, even though the mouth is the primary region of the mask, the model predominantly focuses on the eye region. Similarly, for the last four images where faces are covered by sunglasses, despite the eye being the main region of the mask, the model primarily attends to the mouth region, which aligns with human perception. The results indicate that methods in this paper effectively addresses the occlusion problem.

### 4.4. Confusion matrix analysis

**Figure 7:**
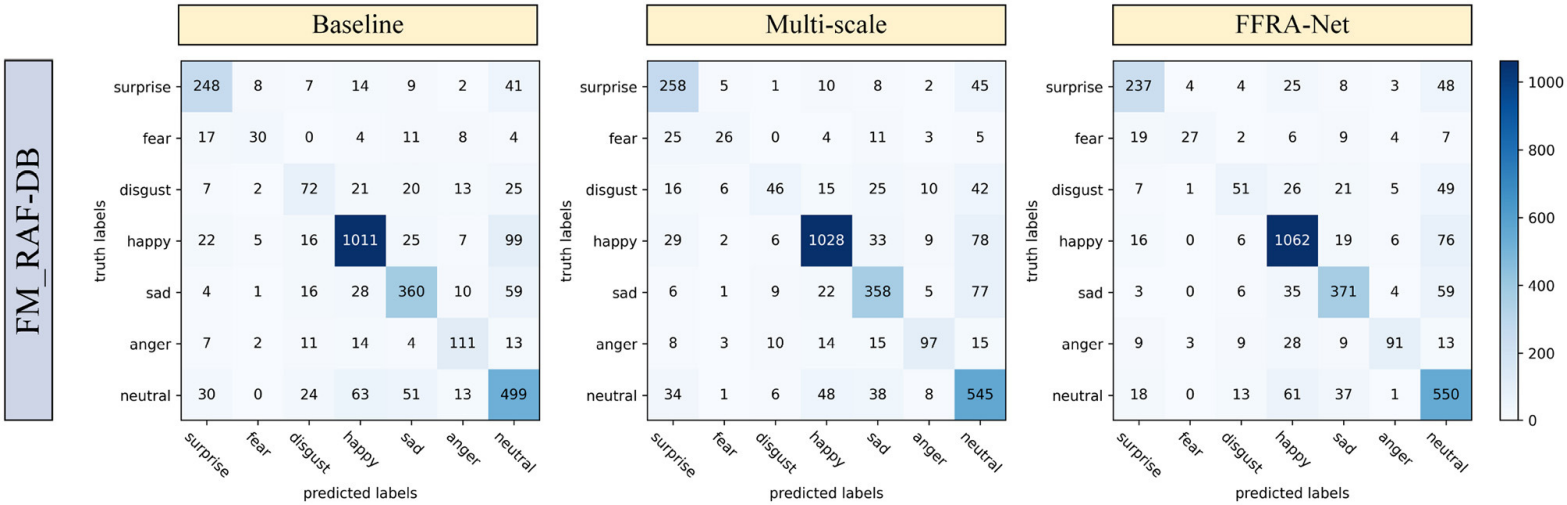
Confusion matrix results for baseline, multi-scale modules and FFRA-Net on the FM_RAF-DB test set.

Confusion Matrix is a valuable tool for evaluating the performance of a classification model. It displays the relationship between the classification model's predictions for different categories and their corresponding true labels, with the table numbers representing the number of predicted samples. The subsection analyze the Confusion Matrix of the baseline, multi-scale module, and FFRA method applied to the test set of the FM_RAF-DB dataset. Figure 7 displays the Confusion Matrix. FFRA Method significantly improves the recognition accuracy of the neutral expression category. Neutral expressions, being states without obvious emotional signals, may lack distinct facial expression features compared to other expression categories. However, FFRA Method can effectively focus on more accurate and relevant features when recognizing neutral expressions, thereby enabling the model to achieve higher recognition accuracy.

### 4.5. Assessment of the model's performance in real-world scenarios

**Figure 8:**
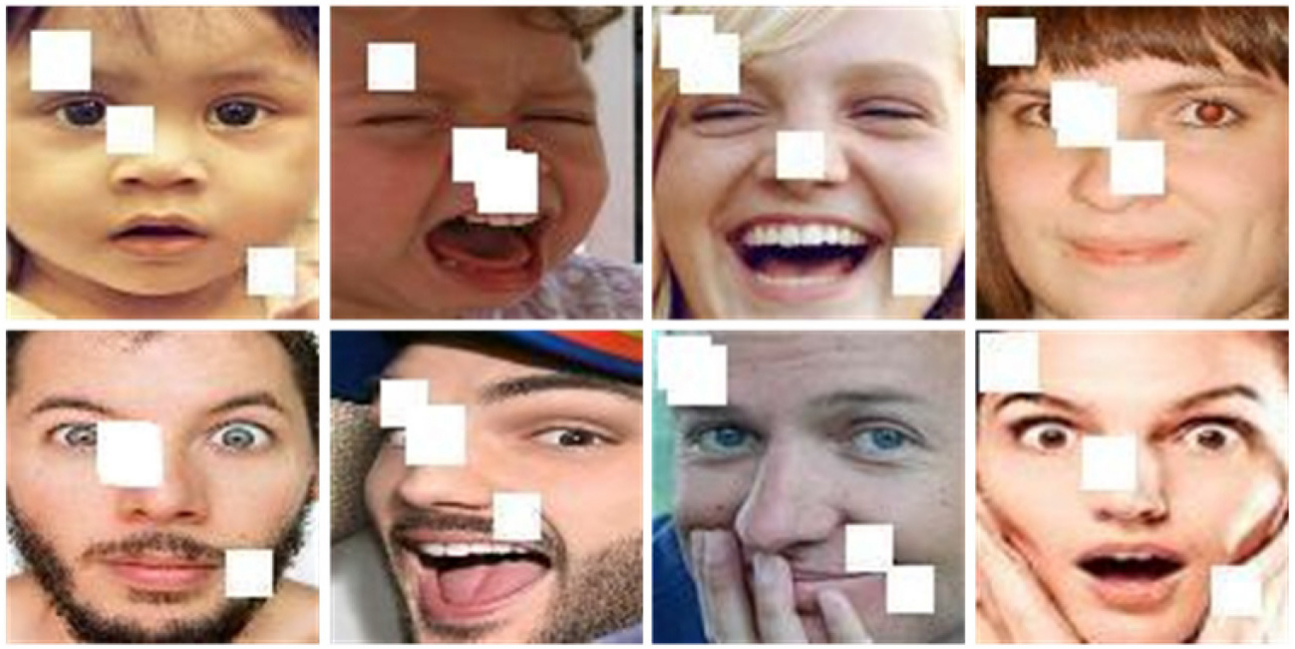
The images were captured from the test set of the RAF-DB dataset, augmented with random occlusion.

To further validate the performance of the FFRA model in real-world environments, the test set of the RAF-DB dataset was added with random occlusion, as depicted in Figure 8. The model achieves an accuracy of 86.43% on this dataset, surpassing the performance of other FER methods listed in Table 3. This demonstrates the outstanding performance of the model in real-world scenarios.

**Table 3:** Comparison of performance with previous FER methods on the test set of the RAF-DB dataset after incorporating random occlusion.

**Method**	**Acc.(%)**
Baseline	81.62
SCN (Wang et al., 2020)	85.78
MA-Net (Zhao et al., 2021)	86.23
**FFRA-Net (Ours)**	**86.43**

The bold values are outcomes from model runs described in this paper.

### 4.6. Comparison with previous results

In this section, FFRA-Net is compared with other state-of-the-art methods using the FM_RAF-DB and SG_RAF-DB datasets. Specifically, VGG-16 (Simonyan and Zisserman, 2014), ResNet-50 (He et al., 2015), and MobileNetv2 (Sandler et al., 2018) are models with larger parameter counts, deeper networks, and lighter weights, respectively, while SCN (Wang et al., 2020) and MA-Net (Zhao et al., 2021) are specifically designed for FER in the wild. The experimental results in Table 4 demonstrate that FFRA-Net outperforms the other FER models in terms of accuracy, showcasing excellent performance.

**Table 4:** Performance comparison (%) with previous methods on FM_RAF-DB and SG_RAF-DB.

**Methods**	**FM_RAF-DB**	**SG_RAF-DB**
VGG-16 (Simonyan and Zisserman, 2014)	73.86	75.81
ResNet-50 (He et al., 2015)	74.32	75.88
MobileNetv2 (Sandler et al., 2018)	73.14	75.46
SCN (Wang et al., 2020)	76.43	77.64
MA-Net (Zhao et al., 2021)	77.64	78.78
**FFRA-Net(Ours)**	**77.87**	**79.50**

The bold values are outcomes from model runs described in this paper.

FFRA method achieves an accuracy of 77.87% on the FM_RAF-DB dataset and 79.50% on the SG_RAF-DB dataset. These results surpass several existing mainstream methods and occluded FER methods. The proposed FFRA-Net in this paper exhibits outstanding performance in recognizing obscured expression images.

The accuracy results of FFRA-Net and other FER models on the RAF-DB and FERPLUS datasets are shown in Table 5. The FFRA method achieves an accuracy of 88.66% on the RAF-DB dataset and 88.97% on the FERPLUS dataset. These results outperform several existing FER methods in the wild. The results demonstrate that the proposed method in this paper exhibits strong generalization ability.

**Table 5:** Performance comparison (%) with previous methods on RAF-DB and FERPLUS.

**Methods**	**RAF-DB**	**FERPLUS**
gACNN (Li et al., 2019)	85.07	-
RAN (Wang K. et al., 2019)	86.90	88.55
SCN (Wang et al., 2020)	87.03	88.01
DACL (Farzaneh and Qi, 2021)	87.78	-
KTN (Li et al., 2021)	88.07	-
MA-Net (Zhao et al., 2021)	88.40	-
RUL (Zhang et al., 2021)	-	88.75
DMUE (She et al., 2021)	-	88.64
SeNet50 (Albanie et al., 2018)	-	88.80
**FFRA-Net(Ours)**	**88.66**	**88.97**

The bold values are outcomes from model runs described in this paper.

FFRA method achieves an accuracy of 88.66% on the RAF-DB dataset and 88.97% on the FERPLUS dataset. These results surpass several existing expression recognition methods. The results show that the method proposed in this paper has a strong generalization ability.

## 5. Conclusion

To solve the problem of occluded FER, a new feature fusion architecture, called FFRA-Net, is proposed, which can learn a rich diversity of global and local features. First, a multi-scale module is proposed to provide diverse global features. Second, an attention-based mechanism local attention module is proposed, which assigns higher weights to important facial regions and smaller weights to irrelevant facial regions. Finally, a feature fusion module is proposed, which uses a weighted approach to fuse global and local features. Extensive experiments on four FER datasets show that this method outperforms the existing FER methods. However, the model requires further optimization in terms of parameter reduction to alleviate computational overhead. A primary area of future research is the investigation of lightweight techniques for occluded FER.

## Data availability statement

The original contributions presented in the study are included in the article/supplementary material, further inquiries can be directed to the corresponding author.

## Author contributions

Data curation: YC. Conceptualization: YC, SL, and DZ. Methodology, software, writing—original draft, and validation: YC and DZ. Formal analysis: YC, SL, and WJ. Supervision: SL. All authors have read and agreed to the published version of the manuscript.

## References

[B1] Albanie S. Nagrani A. Vedaldi A. Zisserman A. (2018). “Emotion recognition in speech using cross-modal transfer in the wild,” in Proceedings of the 26th ACM international conference on Multimedia, pages (New York, NY), 292–301. 10.1145/3240508.3240578

[B2] Barsoum E. Zhang C. Canton-Ferrer C. Zhang Z. (2016). “Training deep networks for facial expression recognition with crowd-sourced label distribution,” in Proceedings of the 18th ACM International Conference on Multimodal Interaction (New York, NY), 279–283. 10.1145/2993148.2993165

[B3] Chen Y. Joo J. (2021). “Understanding and mitigating annotation bias in facial expression recognition,” in 2021 IEEE/CVF International Conference on Computer Vision (ICCV) (Montreal, QC: IEEE), 14960–14971. 10.1109/ICCV48922.2021.01471

[B4] Chen Y. Wang J. Chen S. Shi Z. Cai J. (2019). “Facial motion prior networks for facial expression recognition,” in 2019 IEEE Visual Communications and Image Processing (VCIP) (Sydney, NSW: IEEE), 1–4. 10.1109/VCIP47243.2019.8965826

[B5] Deng J. Guo J. Ververas E. Kotsia I. Zafeiriou S. FaceSoft I. (2020). “Retinaface: Single-shot multi-level face localisation in the wild,” in 2020 IEEE/CVF Conference on Computer Vision and Pattern Recognition (CVPR) (Seattle, WA: IEEE). 10.1109/CVPR42600.2020.00525

[B6] Ding H. Zhou P. Chellappa R. (2020). “Occlusion-adaptive deep network for robust facial expression recognition,” in 2020 IEEE International Joint Conference on Biometrics (IJCB) 1–9 (Houston, TX: IEEE). 10.1109/IJCB48548.2020.9304923

[B7] Farzaneh A. H. Qi X. (2021). Facial expression recognition in the wild via deep attentive center loss,” in 2021 IEEE Winter Conference on Applications of Computer Vision (WACV), 2401–2410 (Waikoloa, HI: IEEE). 10.1109/WACV48630.2021.00245

[B8] Gao S. Cheng M.-M. Zhao K. Zhang X. Yang M.-H. Torr P. H. S. (2019). Res2net: A new multi-scale backbone architecture. IEEE Trans. Patt. Analy. Mach. Intell. 43, 652–662. 10.1109/TPAMI.2019.2938758 31484108

[B9] He K. Zhang X. Ren S. Sun J. (2015). “Deep residual learning for image recognition,” in 2016 IEEE Conference on Computer Vision and Pattern Recognition (CVPR) (Las Vegas, NV: IEEE), 770–778. 10.1109/CVPR.2016.90

[B10] Kim J. H. Poulose A. Han D. S. (2021). The extensive usage of the facial image threshing machine for facial emotion recognition performance. Sensors. 21, 2026. 10.3390/s21062026 33809352 PMC7998952

[B11] Li H. Wang N. Ding X. Yang X. Gao X. (2021). Adaptively learning facial expression representation via c-f labels and distillation. IEEE Trans. Image Proc. 30, 2016–2028. 10.1109/TIP.2021.3049955 33439841

[B12] Li S. Deng W. (2019). Reliable crowdsourcing and deep locality-preserving learning for unconstrained facial expression recognition. IEEE Trans. Image Proc. 28, 356–370. 10.1109/TIP.2018.2868382 30183631

[B13] Li Y. Zeng J. Shan S. Chen X. (2018). “Patch-gated cnn for occlusion-aware facial expression recognition,” in 2018 24th International Conference on Pattern Recognition (ICPR) (Beijing: IEEE), 2209–2214. 10.1109/ICPR.2018.8545853

[B14] Li Y. Zeng J. Shan S. Chen X. (2019). Occlusion aware facial expression recognition using cnn with attention mechanism. IEEE Trans. Image Proc. 28, 2439–2450. 10.1109/TIP.2018.2886767 30571627

[B15] Lucey P. Cohn J. F. Kanade T. Saragih J. M. Ambadar Z. Matthews I. (2010). “The extended cohn-kanade dataset (ck+): A complete dataset for action unit and emotion-specified expression,” in 2010 IEEE Computer Society Conference on Computer Vision and Pattern Recognition-Workshops (San Francisco, CA: IEEE), 94–101. 10.1109/CVPRW.2010.5543262

[B16] Ma R. Zhang R. (2023). Facial expression recognition method based on PSA-YOLO network. Front. Neurorob. 16, 1057983. 10.3389/fnbot.2022.1057983 36733905 PMC9887114

[B17] Marrero-Fernández P. D. Guerrero-Pe na F. A. Tsang I. R. Cunha A. (2019). “Feratt: Facial expression recognition with attention net,” in 2019 IEEE/CVF Conference on Computer Vision and Pattern Recognition Workshops (CVPRW) (Long Beach, CA: IEEE), 837–846. 10.1109/CVPRW.2019.00112

[B18] Pan B. Wang S. Xia B. (2019). “Occluded facial expression recognition enhanced through privileged information,” in Proceedings of the 27th ACM International Conference on Multimedia (New York, NY: ACM), 566–573. 10.1145/3343031.3351049

[B19] Poulose A. Kim J. H. Han D. S. (2021a). “Feature vector extraction technique for facial emotion recognition using facial landmarks,” in 2021 International Conference on Information and Communication Technology Convergence (ICTC) (Jeju Island: IEEE), 1072–1076. 10.1109/ICTC52510.2021.9620798

[B20] Poulose A. Reddy C. S. Kim J. H. Han D. S. (2021b). “Foreground extraction based facial emotion recognition using deep learning xception model,” in 2021 Twelfth International Conference on Ubiquitous and Future Networks (ICUFN) (Jeju Island: IEEE), 356–360. 10.1109/ICUFN49451.2021.9528706

[B21] Proverbio A. M. Cerri A. (2022). The recognition of facial expressions under surgical masks: The primacy of anger. Front. Neurorob. 16, 864490. 10.3389/fnins.2022.864490 35784837 PMC9243392

[B22] Pu T. Chen T. Xie Y. Wu H. Lin L. (2020). “Au-expression knowledge constrained representation learning for facial expression recognition,” in 2021 IEEE International Conference on Robotics and Automation (ICRA) (IEEE), 11154–11161. 10.1109/ICRA48506.2021.9561252

[B23] Sandler M. Howard A. G. Zhu M. Zhmoginov A. Chen L.-C. (2018). “Mobilenetv2: Inverted residuals and linear bottlenecks,” in 2018 IEEE/CVF Conference on Computer Vision and Pattern Recognition (Salt Lake City, UT: IEEE), 4510–4520. 10.1109/CVPR.2018.00474

[B24] She J. Hu Y. Shi H. Wang J. Shen Q. Mei T. (2021). “Dive into ambiguity: Latent distribution mining and pairwise uncertainty estimation for facial expression recognition,” in 2021 IEEE/CVF Conference on Computer Vision and Pattern Recognition (CVPR) (Nashville, TN: IEEE), 6244–6253. 10.1109/CVPR46437.2021.00618

[B25] Simonyan K. Zisserman A. (2014). Very deep convolutional networks for large-scale image recognition. arXiv preprint arXiv:1409.1556. 10.48550/arXiv.1409.1556

[B26] Valstar M. F. Pantic M. (2010). Induced disgust, happiness and surprise : an addition to the mmi facial expression database,” in Proceedings of the 3rd International Workshop on EMOTION (satellite of LREC): Corpora for Research on Emotion and Affect.

[B27] Wang C. Wang S. Liang G. (2019). “Identity- and pose-robust facial expression recognition through adversarial feature learning,” in Proceedings of the 27th ACM International Conference on Multimedia (New York, NY: ACM), 238–246. 10.1145/3343031.3350872

[B28] Wang K. Peng X. Yang J. Lu S. Qiao Y. (2020). “Suppressing uncertainties for large-scale facial expression recognition,” in 2020 IEEE/CVF Conference on Computer Vision and Pattern Recognition (CVPR) (Seattle, WA: IEEE), 6896–6905. 10.1109/CVPR42600.2020.00693

[B29] Wang K. Peng X. Yang J. Meng D. Qiao Y. (2019). Region attention networks for pose and occlusion robust facial expression recognition. IEEE Trans. Image Proc. 29, 4057–4069. 10.1109/TIP.2019.2956143 32011249

[B30] Wang X. Huang J. Zhu J. Yang M. Yang F. (2018). “Facial expression recognition with deep learning,” in International Conference on Internet Multimedia Computing and Service (ACM), 1–4. 10.1145/3240876.3240908

[B31] Wen Z. Lin W.-L. Wang T. Xu G. (2021). Distract your attention: Multi-head cross attention network for facial expression recognition. Biomimetics 8, 199. 10.3390/biomimetics8020199 37218785 PMC10204414

[B32] Xia B. Wang S. (2020). “Occluded facial expression recognition with step-wise assistance from unpaired non-occluded images,” in Proceedings of the 28th ACM International Conference on Multimedia (Seattle, WA: ACM), 2927–2935. 10.1145/3394171.3413773

[B33] Xue F. Wang Q. Guo G. (2021). “Transfer: Learning relation-aware facial expression representations with transformers,” in 2021 IEEE/CVF International Conference on Computer Vision (ICCV) (Montreal, QC: IEEE), 3581–3590. 10.1109/ICCV48922.2021.00358

[B34] Xue F. Wang Q. Tan Z. Ma Z. Guo G. (2022). Vision transformer with attentive pooling for robust facial expression recognition. IEEE Trans. Affec. Comput. 10.1109/TAFFC.2022.3226473

[B35] Zeng J. Shan S. Chen X. (2018). “Facial expression recognition with inconsistently annotated datasets,” in European Conference on Computer Vision (Springer Nature Switzerland), 227–243. 10.1007/978-3-030-01261-8_14

[B36] Zhang F. Zhang T. rong Mao Q. Xu C. (2018). “Joint pose and expression modeling for facial expression recognition,” in 2018 IEEE/CVF Conference on Computer Vision and Pattern Recognition (Salt Lake City, UT: IEEE), 3359–3368. 10.1109/CVPR.2018.00354

[B37] Zhang Q.-L. Yang Y. (2021). “Sa-net: Shuffle attention for deep convolutional neural networks,” in 2021 IEEE International Conference on Acoustics, Speech and Signal Processing (ICASSP) (Toronto, ON: IEEE), 2235–2239. 10.1109/ICASSP39728.2021.9414568

[B38] Zhang Y. Wang C. Deng W. (2021). “Relative uncertainty learning for facial expression recognition,” in Neural Information Processing Systems, eds. M., Ranzato, A., Beygelzimer, Y., Dauphin, P., Liang, J. W., Vaughan (Red Hook, NY: Curran Associates, Inc.), 17616–17627.

[B39] Zhao G. Huang X. Taini M. Li S. Pietikäinen M. (2011). Facial expression recognition from near-infrared videos. Image Vision Comput. 29, 607–619. 10.1016/j.imavis.2011.07.002

[B40] Zhao Z. Liu Q. Wang S. (2021). Learning deep global multi-scale and local attention features for facial expression recognition in the wild. IEEE Trans. Image Proc. 30, 6544–6556. 10.1109/TIP.2021.3093397 34224355

[B41] Zhou B. Khosla A. Lapedriza À. Oliva A. Torralba A. (2015). “Learning deep features for discriminative localization,” in 2016 IEEE Conference on Computer Vision and Pattern Recognition (CVPR) (Las Vegas, NV: IEEE), 2921–2929. 10.1109/CVPR.2016.319

